# Reviewer acknowledgement 2012

**DOI:** 10.1186/1742-4755-10-5

**Published:** 2013-02-27

**Authors:** José M Belizán

**Affiliations:** 1 Institute for Clinical Effectiveness and Health Policy (IECS), Dr Emilio Ravignani 2024, Buenos Aires C1414CPV, Argentina

## Abstract

**Contributing reviewers:**

The editor of *Reproductive Health* would like to thank all our reviewers who have contributed to the journal in Volume 9 (2012). Peer review is essential to the reliable communication of science and we appreciate the effort and generosity of our reviewers who give their time to furthering the excellence and integrity of the journal.

## 

Matilda Aberese Ako

Ghana

Suneth Agampodi

Sri Lanka

Frank Anderson

United States of America

Ragnar Anderson

United States of America

Samuel Anya

Gambia

Berhanemeskel Atsbeha

Ethiopia

Luis Bahamondes

Brazil

James Bamidele

Nigeria

Brahima Bassane

United Kingdom

Eva Bazant

United States of America

Stan Bernstein

United States of America

Nicole Berry

Canada

Sibhatu Biadgilign

Ethiopia

Meghan Bohren

Switzerland

Eugenia Brage

Argentina

Gerard Breart

France

Maurice Bucagu

Switzerland

Pierre Buekens

United States of America

Venkatraman Chandra-Mouli

Switzerland

Pragti Chhabra

India

Anne Cockcroft

South Africa

Mercedes Colomar

Uruguay

Christine Dehlendorf

United States of America

Gonul Dinc

Turkey

Zehra Golbasi

Turkey

Robert Goldenberg

United States of America

Arthur Greil

United States of America

Daniel Grossman

United States of America

Randah Hamadeh

Bahrain

Frans Helmerhorst

Netherlands

Jillian Henderson

United States of America

Justus Hofmeyr

South Africa

Jaco Homsy

United States of America

Maggie Huff-Rousselle

France

Lisa Hurt

United Kingdom

Caio Imaizumi

Brazil

Kirti Iyengar

India

Imtiaz Jehan

Pakistan

Adriana Kaplan

Spain

Torvid Kiserud

Norway

Jane Knight

United Kingdom

Suneeta Krishnan

India

Nicholas Kyei

Ghana

Catherine Kyobutungi

Kenya

Malinee Laopaiboon

Thailand

Mabel Lie

United Kingdom

Jerker Liljestrand

Cambodia

Hui-Sheng Lin

Taiwan

Gunilla Lindmark

Sweden

Enriquito Lu

United States of America

Adriana Gomes Luz

Brazil

Ragaa Mansour

Egypt

Thubelihle Mathole

South Africa

Michael Mbizvo

Switzerland

Samuel Mills

United States of America

Daya Moodley

South Africa

Ann Moore

United States of America

Allisyn Moran

Bangladesh

Fulata Moyo

Switzerland

Twaha Mutyaba

Uganda

Liem T. Nguyen

Viet Nam

Adeline Nyamathi

United States of America

Lemessa Oljira

Ethiopia

Maria José Osis

Brazil

Yutaka Osuga

Japan

Rodolfo Pacagnella

Brazil

Renato Passini

Brazil

Antonino Perino

Italy

Mary Redmayne

New Zealand

Roger Rochat

United States of America

Armando Humberto Seuc

Switzerland

Jai Bhagwan Sharma

India

James Silberzweig

United States of America

Bibha Simkhada

Nepal

Lucy Smith Paintain

United Kingdom

Paul Spiegel

Switzerland

John Stanback

United States of America

Mary Stewart

United Kingdom

Johanne Sundby

Norway

Geeta Swamy

United States of America

Maria Regina Torloni

Brazil

Antoinette Tshefu

Congo, Democratic Republic

Thomas van den Akker

Netherlands

Juan C. Vazquez

Cuba

Ricardo Vernon

Mexico

Elisabeth Vieira

Brazil

Kirsten Vogelsong

United States of America

Demewoz Woldegebreal

Ethiopia

Teshome Woldehanna

Zimbabwe

Paul Young

United States of America

Nina Zamberlin

Argentina

Roger Zerbo

Burkina Faso

